# Neuropsychiatric correlates of olfactory identification and traumatic brain injury in a sample of impulsive violent offenders

**DOI:** 10.3389/fpsyg.2023.1254574

**Published:** 2023-09-29

**Authors:** Vasudeva Murthy Challakere Ramaswamy, Tony Butler, Bianca Ton, Kay Wilhelm, Philip B. Mitchell, Lee Knight, David Greenberg, Andrew Ellis, Val Gebski, Peter William Schofield

**Affiliations:** ^1^School of Medicine and Public Health, University of Newcastle, Newcastle, NSW, Australia; ^2^University of New South Wales, Sydney, NSW, Australia; ^3^Justice Health and Forensic Mental Health Network, Matraville, NSW, Australia; ^4^National Health and Medical Research Council (NHMRC) Clinical Trials Centre, University of Sydney, Camperdown, NSW, Australia; ^5^Neuropsychiatry Service, Hunter New England Mental Health, Newcastle, NSW, Australia

**Keywords:** olfaction, traumatic brain injury, orbitofrontal cortex (OFC), violent behavior, impulsivity

## Abstract

**Background:**

Olfactory deficits have a diverse etiology and can be detected with simple olfactory tests. Key olfactory pathways are located within the frontal and temporal lobes where they are vulnerable to damage due to head trauma. Orbitofrontal cortex (OFC) integrity is important for olfaction and aspects of behavioral regulation. We measured olfactory identification ability in a sample of impulsive violent offenders to determine its associations with history of traumatic brain injury (TBI) and a range of neuropsychiatric indices, including proxies for cognitive ability, impulsivity and social connectedness.

**Methods:**

Male participants were drawn from the ReINVEST study, a randomized controlled trial of sertraline to reduce recidivism in violent impulsive offenders. Criteria for participation in the study included a minimum age of 18 years, a documented history of two or more violent offenses, and a score of 70 or above on the Barratt Impulsiveness Scale (BIS-11). The 16-item “Sniffin sticks” (SS) odor identification test (OI) was administered as were standardized questionnaires regarding previous TBI, additional measures to screen cognition [word reading test of the Wechsler Individuals Achievement Test (WIAT), social connectedness (the Duke Social Support Scale), and a range of other neuropsychiatric conditions or symptoms]. The sample SS scores were compared against published age-specific norms. Univariate and multivariate analyses were performed with SS score (linear regression, within those without hyposmia) or hyposmia (logistic regression) as the outcome.

**Results:**

The mean OI scores were lower than population norms and 16% of participants were classified as hyposmic. Univariate analyses showed associations of SS score with age, WIAT score, impulsivity, TBI and TBI severity, social connectedness, childhood sexual abuse, suicidality and current use of heroin. In multivariate analyses, age, TBI severity and WIAT remained as significant independent predictors of SS score (within the normosmic range) or hyposmia (logistic regression).

**Conclusion:**

Olfactory performance was associated with multiple behavioral phenomena in a pattern that would be consistent with this serving as a proxy for orbitofrontal functioning. As such, OI testing may have utility in further studies of offenders. In future, we will examine whether olfactory score predicts recidivism or response to the administration of sertraline, in terms of reducing recidivism.

## 1. Introduction

The sense of smell plays a crucial role in human behavior and cognition, with the olfactory system engaging brain regions, including the orbitofrontal cortex (OFC), medial temporal lobe, thalamus, and amygdala which also play key roles in behavioral responses and regulation (Sobel et al., 1998; Eslinger et al., 2004; Rolls, 2004). The OFC contains olfactory cortical areas which are particularly important for olfactory identification (OI) and OI testing can serve as a non-invasive measure of its integrity and functioning (Seidman et al., 1992; Kern et al., 2000; Vasterling et al., 2000) given that damage to the OFC has been associated with olfactory dysfunction (OD) (Li et al., 2010; Seubert et al., 2012) as well as behavioral dysregulation and impulsivity (De Guise et al., 2015).

Olfactory dysfunction (OD) is commonly observed in certain neurodegenerative diseases and may occur in the context of neuropsychiatric disorders such as schizophrenia, depression and panic disorder (Ryo et al., 2017; Fóz et al., 2022). Neural pathways for olfactory processing overlap with those important for aspects of executive functioning concentrated in the frontal lobe, and tests of executive function and olfaction have been shown to positively correlate across a broad array of measures, as recently reviewed (Ramaswamy and Schofield, 2022). Indeed, some investigators have suggested that olfactory tests could serve as a rapid screen for cognitive impairment (Jung et al., 2019; Vance et al., 2023).

Traumatic brain injury (TBI) may cause OD, referred to as “post-traumatic olfactory dysfunction” (PTOD) (Howell et al., 2018). PTOD may arise due to damage to the nasal sinuses, peripheral olfactory structures or central, cerebral olfactory structures (Schofield and Doty, 2019). The orbitofrontal and temporal lobes that include principal elements of the olfactory pathways are particularly vulnerable to TBI damage contributing to the association of TBI with OD (Schofield et al., 2014; Arnold et al., 2020; Pellegrino et al., 2021). The intensity and the extent of TBI influence the likelihood of developing PTOD and of its recovery (i.e., less likely when severe) and therefore the presence of OD in the context of a reported past TBI can serve as an indication of possibly greater TBI severity (Konstantinidis et al., 2013; Schofield et al., 2014).

Violent offenders often exhibit impulsivity and aggression and also have high rates of past TBI (Cuomo et al., 2008; Williams et al., 2018); thus it might be anticipated that violent offenders as a group would perform more poorly on olfactory testing than the general population. Although we are not aware of studies that have specifically sought such a finding, several studies offer tangential support for such a hypothesis. Thus, in one study individuals with psychopathic traits, a phenotype overrepresented within offending populations, performed worse than controls on olfactory testing (Mahmut and Stevenson, 2012) and sociopathic behavior (of which impulsive aggression constitutes a form) has been shown to be associated with damage, dysfunction and or relative atrophy of the OFC (Nummenmaa et al., 2021), itself a correlate of olfactory dysfunction, as has been outlined briefly above.

We hypothesized that among a sample of impulsive violent offenders, olfactory performance would be poorer than appropriate population normative scores, on the basis that many such individuals likely manifest behavioral characteristics of OFC dysfunction, in some instances related to past TBI. To the extent that historical indices of TBI severity were available, we also hypothesized that greater severity TBIs would be associated with poorer performance on olfactory testing. Thus, our major objects in this study were, first, to determine if individuals with a violent profile exhibit a distinct olfactory ability compared to the general population and, second, to determine if reported severity of past TBI was reflected in differences in olfactory performance. In previous studies, a number of other factors, including impulsivity, general intelligence and the extent of an individual’s social network, have been shown to be associated with olfactory performance (Danthiir et al., 2001; Meyer et al., 2010; Zou et al., 2016; Herman et al., 2018). Thus our third specific objective was to determine if we could replicate these findings. We hoped that by undertaking this study we could add both to the growing general literature concerning the neuropsychiatric correlates of olfactory test performance, as well as the specific literature relating to evaluations and prediction, pertinent to offending and the criminal justice system.

## 2. Materials and methods

### 2.1. Selection criteria

Participants were men who had been enrolled in a randomized controlled trial designed to investigate the benefits on reduced recidivism of the medication sertraline when administered to impulsive repeat violent offenders (Butler et al., 2021). For this study, only men were included because the parent study from which these data were derived involved phase IV of the clinical trial in which effect of sertraline drug was studied, thus avoiding potential pregnancy complications. In addition, men are more represented in the criminal justice system and there may be a different mechanism of violence in men, so impulsivity was thought to play an important role in violence in men (Denson et al., 2018).

Participants were at least 18 years old and had a prior conviction (with or without imprisonment) for 2 or more violent offenses (e.g., manslaughter, robbery, assault) and scored 70 or higher on the BIS-11 (Barratt et al., 1995). Other requirements were that participants were able to communicate in English, were medically fit and could give informed consent (Butler et al., 2021). With the above selection criteria, 693 participants were selected and out of 693, the sample was reduced to 485 based on individuals who undertook the OI testing.

### 2.2. Data collection

The study’s participants were recruited from referrals made by magistrates and attorneys in the New South Wales regions of Sydney Metropolitan, Central Coast, Western Sydney, and Hunter areas. Under the direction of New South Wale’s corrective services, participants were also selected from community service orders. Other passive recruiting strategies include word-of-mouth, self-referral, a free contact number, study fliers accessible at courts and the study website^1^ (Butler et al., 2021).

### 2.3. Olfactory identification testing

Sniffin sticks: This test of olfaction uses a felt-tipped pen that is impregnated with different odors such that removal of the cap will release the odor. In its most complete form, it allows for the evaluation of three aspects of olfactory functioning—odor identification, odor discrimination, and olfactory thresholds, however, the use of a single measure, most often odor identification as in the current study, is a convenient option to obtain meaningful and valid data (Hummel et al., 1997). The 16-item odor identification test, one of the three components of the “Sniffin sticks” (SS) battery of component tests (Hummel et al., 1997) uses 16 pens, each with a different odor to test olfaction. The subject is asked to identify which of four possible odors (written on cards) the test odor most resembles (Hummel et al., 1997). A total SS score based on OI of less than the 10th percentile for men for respective age categories was used for the determination of hyposmia (Oleszkiewicz et al., 2019).

### 2.4. Traumatic brain injury

Participants were asked to complete the medical history questionnaire which included a questionnaire on past TBI. Participants were asked: “Have you ever had a head injury where you passed out or had a ‘blackout?’ and how often have you experienced a head injury?” Up to five separate episodes of traumatic brain injury were recorded and participants were asked to report the TBI in order of severity. The first reported TBI was the most severe, followed by the second most severe and the third most severe. The traumatic brain injury questionnaire included additional questions: how long were you unconscious (blackout)? When did this happen? Other additional information such as did they suffer a skull fracture or bleeding on the head or surgical procedure on the head?

### 2.5. Demographics

Details related to age, gender, ethnicity, marital status, children, education, employment, and accommodation were sought.

### 2.6. Behavioral and psychiatric measures

Assessments included the BIS-11 and the Eysenck Impulsivity Questionnaire (EIQ) self-assessment questionnaires of impulsive personality. BIS-11 is a 30-item questionnaire that assesses trait of impulsiveness and EIQ is a 54-item questionnaire that assesses impulsive personality. The Anger Irritability and Aggression Questionnaire (AIAQ) is a 42-item questionnaire and was used to measure the subjective level of anger and aggression in recent 2 weeks and the State-Trait Anger Expression Inventory (STAXI-2) is a 57-item questionnaire that was used to measure the subjective level of anger in different situations. The Beck Depression Inventory-II (BDI-II) is a 21-item questionnaire and was used to examine symptoms in the past week that may indicate a mood disorder. The Alcohol Use Disorders Identification Test (AUDIT) measured alcohol consumption in the 12 months before incarceration. The AUDIT identifies safe, harmful and hazardous levels of alcohol consumption. The Kessler Psychological Distress Scale (K-10) is a 10-item questionnaire that provides a global measure of distress, based on questions about anxiety and depressive symptoms in the past 4 weeks. The International Personality Disorder Examination (IPDE) was used to categorize participants as having Impulsive Personality Disorder, Dissocial Personality Disorder, and Borderline Personality Disorder. Details of substance abuse were collected as well as information on suicide attempts, self-harm and sexual abuse, as part of the routine reception interview. The single word reading test from the Wechsler Individual Achievement Test was also administered to provide a proxy measure for reading ability/intelligence (Wechsler, 2001). In this test, points are scored by correct pronunciation of words, sequentially presented, of escalating rarity and difficulty.

### 2.7. Social measures

The Duke Social Support Scale is a 11-item questionnaire that provided information on only single component of social interaction and the Quality of Life Short Form Questionnaire (SF-12) is a 12-item questionnaire that assessed health-related quality of life.

### 2.8. Procedure

The BIS-11 was administered by research nurse when the first contact is made with participant. Participants who met the inclusion criteria and had a BIS-11 score of at least 70 underwent medical assessment. The study assessments were conducted in person. The inclusion/exclusion criteria were verified by clinical assessment and review of the court documentation (i.e., police fact sheets and criminal records). The clinical assessment included a comprehensive psychiatric assessment, physical examination and blood testing full blood count, liver function test, thyroid function test and kidney function test (providing the participant consented to the blood test). Participants were generally assessed over one session (approximately 3 h in duration) and then followed up fortnightly for 6 weeks and then monthly. They were followed up at other intervals depending on clinical need. Assessment sessions were conducted either in a private room hire (during COVID), court complexes or community corrections. Follow up appointments were conducted at locations which were convenient for the participant and safe for the staff (i.e., community corrections, libraries, etc.). For some reviews, particularly during COVID, telehealth was utilized, however, assessments were always face to face. The following tests were administered after the medical assessment: the EIQ, STAXI-2, BDI, K-10, SF-12, AIAQ, AUDIT, IPDE, Duke social support, TBI history, WIAT, and substance misuse.

### 2.9. Statistical analyses

Initial analyses compared the sample characteristics, and psychological and functional measures between participants who took SS and those who did not. In subsequent analyses, involving the sample with SS data, we examined sample characteristics, presence of TBI, time length of loss of consciousness (LOC), time of occurrence, severity of head injury, symptom following head injury, and overall response distribution of the psychological and functional measures. Hosmer–Lemeshow goodness of fit test based on chi-square formulation was used to investigate the association of the presence of TBI with sample characteristics, personality disorder, psychiatric assessment, and substance abuse. This procedure was repeated with respect to different study outcomes, including duration of LOC, time of occurrence, and LOC of participants who reported only one episode of TBI. Fisher’s exact test was used when the expected count assumption of the chi-square test was not achieved. Meanwhile, one-way ANOVA (equal variance not assumed) with *post-hoc* test (Tukey), when the pairwise comparison is necessary, was performed to investigate the study outcome with continuous numerical covariates including demographic characteristics, and psychological and functional measures. Two-way ANOVA (Type 3) was computed to investigate the association of mean Sniffin stick score and age category by hyposmia and normal olfaction group. *Post-hoc* test (Bonferroni) was used for pairwise comparison. Linear regression modeling was computed to predict the Sniffin stick score. Forward variable selection using Akaike Information Criterion was computed to determine salient variables. Overall linearity and linearity of each selected variable was checked with residual plot. Normality was determined with histogram of residuals. All possible two-way interaction terms were checked for significant relationship between covariates. Multicollinearity was checked with variance inflation factor. R-square was used to determine the percentage of variance explained for the prediction model. Logistic regression modeling was computed to predict hyposmia. Forward variable selection using Akaike Information Criterion was computed to determine salient variables. Overall linearity and linearity of each selected numerical variable was checked with residual plot. All possible two-way interaction terms were checked for significant relationship between covariates. Multicollinearity was checked with variance inflation factor. Model fit was determined using Hosmer and Lemeshow goodness of fit test. Cook’s distance was used to check for outliers. R-square (McFadden method) was used to determine the percentage of variance explained for the prediction model All statistical procedures were two-sided and computed on R4.3.3. All results are interpreted at 5% significance level. To help clarify relationships between TBI, impulsivity and olfaction we examined these data in tabular form and then performed simple and logistic regression analyses, with olfaction or hyposmia as the dependent variable with these and additional explanatory variables.

## 3. Results

All data for the present analyses were obtained from participants at the baseline of the study, before any exposure to sertraline. Data from 693 individuals was used for the initial analysis, however, for a variety of reasons, including difficulties relating to the COVID epidemic, 208 did not undergo OI testing. Differences between those who did not and the 485 who did get SS were few: the former were somewhat more impulsive, more likely to have been suspended, had more suicidality, and reported more alcohol and substance use (Supplementary Tables 1, 2).

In the 5 years prior to their recruitment to the study, participant offending histories comprised the following: 98% had been found guilty of any offense and 48% had been in custody; 63% had committed an act of domestic violence and 58% an act of non-domestic violence; 51% had committed property damage and 87% had committed other offenses not included in the above categories of offending.

When SS scores of the 485 were compared against normative data for age, the offender sample as a group demonstrated significantly lower olfactory performance in the age category 21–30, 31–40, and 41–50 (Oleszkiewicz et al., 2019; Table 1).

**TABLE 1:** Sniffin stick score comparison of offender population vs. population norms.

Age groups (years)	Sniffin’ sticks total score						*t* value (df)	*p*-value
	**Impulsive violent offenders (Present study)**			**Population norm (Oleszkiewicz et al., 2019)**				
	***N***	**Mean**	**SD**	***N***	**Mean**	**SD**		
11–20	55	12.60	1.75	439	12.85	1.81	−0.97 (492)	0.330
21–30	202	12.65	1.84	857	13.63	1.72	−7.19 (1,057)	**< 0.001**
31–40	130	12.75	1.81	282	13.63	1.60	−4.97 (410)	**< 0.001**
41–50	79	12.28	2.21	199	13.25	1.85	−3.73 (276)	**< 0.001**
51–60	17	12.47	1.74	221	12.85	2.05	−0.74 (236)	0.460
61–70	02	10.50	0.71	141	12.20	2.55	−0.94 (141)	0.350
Total	485			2,139				

Bold values indicate statistical significance at 0.05 level.

Table 2 presents the demographic features of the SS sample stratified by hyposmia and normal olfaction. About 67% of the study participants were between 18 to 35 years old. Most (85%) were of non-Aboriginal descent and living with their parents, partners, or relatives (86%). Half were never married and 45% of participants with hyposmia had been unemployed for at least 6 months.

**TABLE 2:** Demographic data by Sniffin sticks category (*n* = 485).

Variables	Overall (*n* = 485)	Hyposmia (*n* = 78)	Normal (*n* = 407)	
	***n* (%)**	***n* (%)**	***n* (%)**	***P*-value (a)**
**Age (years)**				0.167
18–35	326 (67.0%)	46 (59.0%)	280 (69.0%)	
36–55	149 (31.0%)	31 (40.0%)	118 (29.0%)	
> 55	10 (2.1%)	1 (1.3%)	9 (2.2%)	
**Ethnicity**				0.154
Not aboriginal and or Torres Strait Islander	410 (85.0%)	62 (79.0%)	348 (86.0%)	
Aboriginal	67 (14.0%)	13 (17.0%)	54 (13.0%)	
Torres Strait Islander	6 (1.2%)	2 (2.6%)	4 (1.0%)	
Both aboriginal and/or Torres Strait Islander	2 (0.4%)	1 (1.3%)	1 (0.2%)	
**Marital Status**				0.165 (b)
Single (never married)	248 (51.0%)	43 (55.0%)	205 (50.0%)	
Regular partner	134 (28.0%)	24 (31.0%)	110 (27.0%)	
Married	68 (14.0%)	7 (9.0%)	61 (15.0%)	
Separated	25 (5.2%)	1 (1.3%)	24 (5.9%)	
Divorced	9 (1.9%)	3 (3.8%)	6 (1.5%)	
Number of children, mean (SD)	1.72 (1.97)	1.83 (2.10)	1.70 (1.95)	0.867 (c)
Age at leaving school, mean (SD)	15.69 (1.72)	15.36 (1.44)	15.75 (1.77)	**0.044** (c)
Number of schools changed before dropping out, mean (SD)	4.43 (5.43)	3.86 (2.78)	4.54 (5.80)	0.459 (c)
Number of times suspended from school, mean (SD)	11.00 (19)	11.76 (18.51)	10.69 (19.37)	0.689 (c)
Number of times expelled from school, mean (SD)	2.11 (2.53)	2.13 (3.58)	2.11 (2.29)	0.183 (c)
Wechsler Individual Achievement Test score, mean (SD)	116.00 (16.00)	115.61 (16.12)	116.96 (16.46)	0.347 (c)
**Education**				0.602 (b)
Never attended school	3 (0.6%)	2 (2.6%)	1 (0.2%)	
Completed primary school only	12 (2.5%)	2 (2.6%)	10 (2.5%)	
Left school with no qualification	156 (32.0%)	27 (35.0%)	129 (32.0%)	
School certificate	177 (36.0%)	26 (33.0%)	151 (37.0%)	
HSC/VCE/leaving certificate	52 (11.0%)	8 (10.0%)	44 (11.0%)	
College certificate/diploma	14 (2.9%)	3 (3.8%)	11 (2.7%)	
Technical or trade qualification	61 (13%)	9 (12%)	52 (13%)	
Degree/tertiary education	9 (1.9%)	1 (1.3%)	8 (2.0%)	
**Employment**				0.334
Unemployed for at least 6 months	186 (38.0%)	35 (45.0%)	151 (37.0%)	
Employed within last 6 months	298 (61.0%)	43 (55.0%)	255 (63.0%)	
**Accommodation**				0.671
Live alone	63 (13.0%)	11 (14.0%)	52 (13.0%)	
Partner	131 (27.0%)	20 (26.0%)	111 (27.0%)	
Mother/father	139 (29.0%)	21 (27.0%)	118 (29.0%)	
Mother/father and partner	12 (2.5%)	4 (5.1%)	8 (2.0%)	
Other relatives	138 (28.0%)	22 (28.0%)	116 (29.0%)	
**Have you ever attended any special schools/classes?**				0.099
No	312 (65.0%)	44 (56.0%)	268 (66.0%)	
Yes	171 (35.0%)	34 (44.0%)	137 (34.0%)	
**Have you ever been expelled from a school?**				0.969
No	292 (60.0%)	47 (60.0%)	245 (60.0%)	
Yes	191 (40.0%)	31 (40.0%)	160 (40.0%)	
**Have you ever been suspended from a school?**				0.152
No	124 (26.0%)	15 (19.0%)	109 (27.0%)	
Yes	358 (74.0%)	63 (81.0%)	295 (73.0%)	

(a) Hosmer–Lemeshow goodness of fit test. (b) Fisher’s exact test. (c) One-way ANOVA (equal variance not assured; Bonferroni *post-hoc* test where significant). SD, Standard deviation. Bold values indicate statistical significance at 0.05 level.

### 3.1. Psychological and functional measures of hyposmia within the sample

Table 3 presents the results of the association of psychological and functional measures and TBI, by SS category (hyposmia or normal olfaction) in the SS sample. Relative to men with normal olfaction, those with hyposmia had significantly higher impulsiveness (BIS-11), anger expression-in, LOC, and more childhood sexual abuse, suicidal attempts and self-harm. When individual drugs were analyzed, current users of heroin were likely to be hyposmic. Surprisingly, a lower mean score on The Beck Depression Inventory was present for the hyposmic group, however, scores on this measure were mostly within the normal or minimally depressed range. A total of 269 individuals (55%) gave a history of any TBI associated with LOC. Although not statistically significant, more individuals with any past TBI with LOC (64%) had hyposmia than those who denied any TBI with LOC, but hyposmia was significant and more likely among those who reported a TBI with LOC > 30 mins. This relationship was also present within the sample who reported only a single TBI with LOC (Table 4).

**TABLE 3:** Participant psychological and functional measures by Sniffin sticks category.

Variables	Overall (*n* = 485)	Hyposmia (*n* = 78)	Normal (*n* = 407)	
	**Mean (SD)**	**Mean (SD)**	**Mean (SD)**	***P*-value (a)**
**Barratt Impulsiveness Scale**	85.00 (10.00)	86.92 (9.70)	84.05 (9.85)	**0.016**
**Eysenck Impulsivity Questionnaire**				
Impulsiveness	13.30 (3.90)	13.68 (3.90)	13.28 (3.89)	0.382
Venturesomeness	11.14 (3.13)	11.45 (3.17)	11.08 (3.12)	0.249
Empathy	11.50 (3.70)	11.00 (3.80)	11.56 (3.65)	0.203
**Anger, irritability, and assault questionnaire**				
Irritability	18.00 (6.00)	17.76 (6.49)	18.58 (5.89)	0.358
Anger lability	12.00 (5.00)	12.59 (4.59)	11.86 (5.04)	0.197
Direct assault	17.00 (7.00)	17.47 (7.06)	17.11 (7.00)	0.547
Verbal assault	17.00 (5.10)	17.67 (5.19)	16.82 (5.06)	0.070
Indirect assault	7.20 (3.60)	7.14 (3.70)	7.20 (3.58)	0.972
**Beck Depression Inventory**	11.00 (8.00)	9.17 (8.90)	11.45 (8.09)	**0.004**
**Kessler Psychological Distress Scale**				
k10	15.00 (9.00)	14.09 (8.11)	15.43 (8.91)	0.276
**Duke Social Support Scale**				
Duke score	24.20 (4.60)	23.71 (4.96)	24.30 (4.51)	0.507
**Quality of Life Short Form Questionnaire**				
Physical component	54.0 (7.00)	53.06 (6.88)	53.65 (6.88)	0.316
Mental component	40.00 (12.00)	40.76 (12.03)	40.34 (12.40)	0.792
**State-Trait Anger Expression Inventory-2**				
State anger	16.70 (4.50)	16.65 (5.18)	16.71 (4.36)	0.521
Trait anger	25.00 (7.00)	25.00 (6.79)	24.84 (6.59)	0.826
Anger expression-out	20.60 (5.00)	21.06 (4.98)	20.51 (5.05)	0.318
Anger expression-in	19.10 (4.30)	20.14 (4.51)	18.89 (4.24)	**0.014**
Anger control-out	17.40 (4.60)	17.99 (4.81)	17.25 (4.51)	0.150
Anger control-in	18.20 (4.70)	18.65 (5.51)	18.09 (4.57)	0.352
Anger expression-index	52.00 (14.00)	52.56 (14.18)	52.06 (13.42)	0.973
**Alcohol Use Disorders Identification Test**	12.00 (9.00)	10.64 (8.21)	11.92 (8.80)	0.255
**TBI**				0.094 (b)
No	216 (44.5%)	28 (36.0%)	188 (46.0%)	
Yes	269 (55.5%)	50 (64.0%)	219 (54.0%)	
**Number of TBI**				0.830 (b)
1 TBI	132 (27.2%)	26 (52.0%)	106 (48.0%)	
2 TBI	52 (10.7%)	10 (20.0%)	42 (19.0%)	
> 2 TBI	85 (17.5%)	14 (28.0%)	71 (32.0%)	
**Severity based on LOC** *				**0.047 (b)**
Less than 30 min	205 (42.3%)	34 (67.0%)	171 (74.0%)	
More than 30 min	36 (7.4%)	11 (24.0%)	25 (13.0%)	
**Recency of last TBI** *				
Less than 2 years	37 (7.6%)	3 (3.8%)	34 (8.4%)	0.078 (b)
More than 2 years	232 (47.8%)	47 (94.0%)	185 (84.0%)	
**International Personality Disorder Examination**				
Borderline	219 (45.2%)	32 (41.0%)	187 (46.0%)	0.424 (b)
Impulsive	350 (72.2%)	54 (69.0%)	296 (73.0%)	0.528 (b)
Dissocial	207 (42.6%)	32 (41.0%)	175 (43.0%)	0.747 (b)
**Self-harm/Suicide and past sexual abuse**				
suicidal ideation	239 (49.3%)	37 (47.0%)	202 (50.0%)	0.722 (b)
suicidal attempt	107 (22.1%)	18 (23.0%)	89 (22.0%)	0.813 (b)
self-harm or Injury	113 (23.3%)	17 (22.0%)	96 (24.0%)	0.732 (b)
childhood sexual abuse	152 (31.3%)	33 (42.0%)	119 (29.0%)	**0.023** (b)
**Substance abuse (current)**				
Yes	407 (83.9%)	66 (85.0%)	341 (84.0%)	0.855 (b)
No	78 (16.1%)	12 (15.0%)	66 (16.0%)	
**Substance abuse (former)**				
Yes	401 (82.7%)	69 (88.0%)	332 (82.0%)	0.141 (b)
No	84 (17.3%)	9 (12.0%)	75 (18.0%)	
**Heroin**				**0.014 (b)**
Current	12 (2.47%)	7 (29.0%)	5 (8.2%)	
Former	71 (14.6%)	16 (67.0%)	55 (90.0%)	
Never	2 (0.4%)	1 (4.2%)	1 (1.6%)	

*Looking at participants who reported severe TBI. (a) Hosmer–Lemeshow goodness of fit test. (b) Fisher’s exact test. SD, Standard deviation. Bold values indicate statistical significance at 0.05 level.

**TABLE 4:** Participant psychological and functional measures by Sniffin sticks category in participants who reported only one episode of TBI.

Variables	Overall (*n* = 132)	Hyposmia (*n* = 26)	Normal (*n* = 106)	
	**Mean (SD)**	**Mean (SD)**	**Mean (SD)**	***P*-value (a)**
**Barratt Impulsiveness Scale**	85.00 (10.00)	87.77 (7.77)	84.48 (10.39)	0.051
**Eysenck Impulsivity Questionnaire**				
Impulsiveness	13.40 (3.90)	13.69 (4.09)	13.30 (3.85)	0.603
Venturesomeness	11.29 (2.73)	11.85 (3.09)	11.15 (2.63)	0.110
Empathy	11.30 (3.40)	10.58 (3.49)	11.43 (3.38)	0.195
**Anger, irritability, and assault questionnaire**				
Irritability	18.70 (5.70)	18.92 (6.47)	18.66 (5.53)	0.843
Anger lability	12.30 (4.70)	12.46 (4.40)	12.31 (4.75)	0.945
Direct assault	18.00 (7.00)	19.50 (5.62)	17.79 (7.37)	0.358
Verbal assault	17.50 (4.70)	18.92 (3.91)	17.09 (4.86)	0.068
Indirect assault	7.60 (3.90)	7.96 (3.92)	7.50 (3.88)	0.528
**Beck Depression Inventory**	11.00 (9.00)	8.19 (9.59)	11.63 (8.52)	**0.017**
**Kessler Psychological Distress Scale**				
k10	14.00 (9.00)	13.19 (8.19)	14.73 (8.68)	0.438
**Duke Social Support Scale**				
Duke score	24.60 (4.20)	23.54 (4.71)	24.80 (4.07)	0.292
**Quality of Life Short Form Questionnaire**				
Physical component	53.40 (5.90)	52.41 (5.45)	53.60 (5.96)	0.138
Mental component	42.00 (12.00)	41.46 (12.16)	41.53 (12.61)	0.954
**State-Trait Anger Expression Inventory-2**				
State anger	16.67 (3.97)	17.15 (4.19)	16.55 (3.93)	0.496
Trait anger	25.00 (6.00)	26.58 (6.66)	24.54 (6.41)	0.153
Anger expression-out	21.40 (5.20)	22.88 (4.81)	21.07 (5.24)	0.065
Anger expression-in	19.00 (4.10)	21.08 (4.72)	18.53 (3.83)	**0.006**
Anger control-out	17.60 (4.50)	17.58 (3.83)	17.56 (4.68)	0.863
Anger control-in	18.60 (4.70)	19.23 (5.62)	18.47 (4.49)	0.652
Anger expression-index	52.00 (13.00)	55.15 (13.41)	51.57 (12.90)	0.250
**Alcohol Use Disorders Identification Test**	11.00 (9.00)	11.19 (8.27)	11.16 (8.90)	0.884
**TBI Severity based on LOC**				**0.047 (b)**
Less than 30 min	100 (84.0%)	15 (57.6%)	85 (80.0%)	
More than 30 min	19 (16.0%)	7 (26.9%)	12 (11.3%)	
**Recency of last TBI**				0.123 (b)
Less than 2 years	20 (15.2%)	1 (3.8%)	19 (18.0%)	
More than 2 years	112 (84.8%)	25 (96.0%)	87 (82.0%)	
**International Personality Disorder Examination**				
Borderline	52 (39.4%)	10 (38.0%)	42 (40.0%)	0.914 (b)
Impulsive	96 (72.7%)	20 (77.0%)	76 (72.0%)	0.592 (b)
Dissocial	50 (37.9%)	10 (38.0%)	40 (38.0%)	0.946 (b)
**Self-harm/Suicide and past sexual abuse**				
suicidal ideation	63 (47.7%)	11 (42.0%)	52 (49.0%)	0.537 (b)
suicidal attempt	26 (19.6%)	8 (31.0%)	18 (17.0%)	0.113 (b)
self-harm or injury	28 (21.2%)	5 (19.0%)	23 (22.0%)	0.783 (b)
childhood sexual abuse	43 (32.5%)	12 (46.0%)	31 (29.0%)	0.099 (b)
**Substance abuse (Current)**				
Yes	104 (78.8%)	21 (81.0%)	83 (78.0%)	0.783 (b)
No	28 (21.2%)	5 (19.0%)	23 (22.0%)	
**Substance abuse (Former)**				
Yes	104 (78.8%)	21 (81.0%)	83 (78.0%)	0.783 (b)
No	28 (21.2%)	5 (19.0%)	23 (22.0%)	

(a) Hosmer–Lemeshow goodness of fit test. (b) Fisher’s exact test. SD, Standard deviation. Bold values indicate statistical significance at 0.05 level.

Table 4 showed results from participants who reported only one episode of TBI, and the associations of psychological and functional measures with the Sniffin stick category. Participants with single episode TBI are selected for this table to eliminate the bias of second TBI. Hyposmia was significantly associated with depression, LOC and anger expression-in. Those with hyposmia had significantly lower depression and higher anger expression-in scores than those with normal olfaction. To help better understand and depict the relationships between TBI, impulsivity and olfaction, we created tables that examined impulsivity and olfaction scores according to TBI history (Table 5).

**TABLE 5:** Sniffin stick score and Barratt Impulsivity score by TBI status.

With TBI and without TBI				
	Overall (485)	TBI no (216)	TBI yes (269)	*P*-valve
Sniffin stick score, mean (SD)	12.59 (1.89)	12.76 (1.84)	12.46 (1.91)	0.051
Barratt Impulsiveness Scale, mean (SD)	85.00 (10.00)	84.09 (10.10)	84.85 (9.69)	0.309
**LOC with severe TBI**				
	Brief LOC (*n* = 176)	> 10 M (*n* = 29)	> 30 M (*n* = 36)	*P*-value
Sniffin stick score, mean (SD)	12.65 (1.84)*	12.48 (1.35)	11.53 (2.31)*	**0.019**
Barratt Impulsiveness Scale, mean (SD)	85.30 (10.06)	84.45 (8.92)	84.33 (9.47)	0.912
**LOC with only one episode of TBI**				
	Brief LOC (*n* = 83)	> 10 M (*n* = 17)	> 30 M (*n* = 19)	*P*-value
Sniffin stick score, mean (SD)	12.98 (1.75)*	12.47 (1.59)	11.21 (2.70)*	**0.009**
Barratt Impulsiveness Scale, mean (SD)	85.90 (10.39)	85.24 (10.10)	83.21 (8.26)	0.671
**Number of TBI**				
	1 TBI (132)	2 TBI (52)	> 2 TBI (85)	*P*-value
Sniffin stick score, mean (SD)	12.57 (2.07)	12.25 (1.75)	12.41 (1.75)	0.213
Barratt Impulsiveness Scale, mean (SD)	85.13 (9.99)	81.29 (8.66)*	86.60 (9.33)*	**0.004**

*Significant pairwise comparison (Bonferroni method). One-way ANOVA (equal variance not assured; Bonferroni *post-hoc* test where significant). Bold values indicate statistical significance at 0.05 level.

While the severity of TBI injury was associated with the SS score, it was not associated with the impulsivity score. Conversely, the TBI number was not associated with olfaction but was with impulsivity.

Table 6 shows the results of univariate and multivariate linear regression analyses limited to the subsample of men with normal olfaction. As expected, age, Duke Social Support, WIAT, BIS, TBI severity and cognitive ability were each associated with SS in the univariate analyses. In the multivariate model, the retained significant variables were age, WIAT score, and TBI severity, with BIS borderline significant at 0.063. Univariate logistic regression revealed that TBI severity, TBI frequency, BIS and WIAT score were significantly associated with hyposmia (Reference group = Normal olfaction). However, after adjusting for confounding factors, multiple logistic regression discovered that age (36 to 55 group), Moderate and Severe TBI, and WIAT score were significant predictors of hyposmia. The final model accounts for 6.7% (McFadden) of the variance explained for Sniffin sticks score (Model fit chi-square = 2.68, df = 8, *p* = 0.952) (Table 7).

**TABLE 6:** Univariate and multivariate analysis in normal olfaction group.

Linear regression				Multiple linear regression (*R*^2^ = 0.099)		
	**B**	**95% CI**	***p*-value (*R*^2^)**	**Adj. B**	**95% CI**	***p*-value**
**Age (years)**			**0.025 (0.049)**			
18–35 (reference)	–	–		–	–	
36–55	−0.37	−0.74, −0.01		−0.51	−0.88, −0.14	**0.008**
> 55	−1.20	−2.40, −0.05		−0.64	−1.90, 0.58	0.300
**TBI severity**			**0.001 (0.045)**			
No TBI (reference)	–	–		–	–	
Mild TBI (< 30 min)	−0.14	−0.50, 0.22		−0.15	−0.51, 0.20	0.400
Moderate/severe TBI (> 30 min)	−1.20	−1.90, −0.58		−1.10	−1.80, −0.45	**0.001**
**Barratt Impulsiveness Scale**	−0.03	−0.04, −0.01	**0.003 (0.055)**	−0.02	−0.03, 0.00	0.065
**Duke Social Support Scale**	0.04	0.01, 0.08	**0.023 (0.050)**	0.04	0.00, 0.07	0.068
**WIAT score**	0.02	0.01, 0.03	**< 0.001 (0.069)**	0.02	0.01, 0.03	**< 0.001**

B, regression coefficient; CI, confidence interval; Adj., adjusted; *R*^2^, coefficient of determination. Bold values indicate statistical significance at 0.05 level.

**TABLE 7:** Univariate and multivariate analysis predicting hyposmia group.

Reference = normal olfaction	Logistic regression			Multiple logistic regression (*R*^2^ = 0.067)		
	**OR**	**95% CI**	***p*-value**	**Adj. OR**	**95% CI**	***p*-value**
**Age (years)**			0.167			
18–35 (reference)	–	–		–	–	
36–55	1.60	0.96, 2.64		1.96	1.12, 3.41	**0.017**
> 55	0.68	0.04, 3.72		0.00	0.00, 0.00	> 0.999
**TBI Severity**			**0.042**			
No TBI (reference)	–	–		–	–	
Mild TBI (< 30 min)	1.34	0.78, 2.31		1.36	0.77, 2.42	0.300
Moderate/severe TBI (> 30 min)	2.95	1.28, 6.57		2.83	1.12, 6.83	**0.023**
**Barratt Impulsiveness Scale**	1.03	1.00, 1.05	**0.020**	1.02	0.99, 1.05	0.200
**Duke Social Support Scale**	0.97	0.92, 1.03	0.298	0.97	0.91, 1.03	0.300
**WIAT score**	0.98	0.97, 1.00	**0.008**	0.98	0.97, 1.00	**0.009**

OR, odds ratio; Adj., adjusted; CI, confidence interval; *R*^2^, coefficient of determination.

## 4. Discussion

Participants of this study were all impulsive men who had committed violent offenses. There were very high rates of TBI, personality disorder, suicidality and substance abuse. The main findings with respect to olfaction were, as we hypothesized, that the sample overall had lower mean SS scores compared to the general population norms, with 16% of participants classified as having hyposmia. Our second hypothesis was also supported: greater TBI severity on self-report (i.e., LOC > 30 min) was associated with poorer scores on olfactory testing, both in univariate and in multivariate analyses adjusted for other factors. With respect to our third objective, impulsivity scores were (inversely) associated with olfactory performance in group comparisons, and univariate and multivariate analyses and we found associations between olfactory performance and a cognitive screen (the WIAT), and differences in grouped comparisons (hyposmia vs. normosmia) for depressive symptoms, anger, and childhood sexual abuse.

The finding that the mean performance on the olfactory identification ability of the sample was lower than population norms has multiple possible explanations. While it is well-established that olfactory dysfunction may arise as a consequence of injury or disease there is now evidence that cognitive test performance is intrinsically associated with olfaction, leading to positive correlations of cognitive testing with olfactory performance in the presumptive absence of underlying pathology (Danthiir et al., 2001; Meyer et al., 2010; Dahmani et al., 2018; Gellrich et al., 2021). Within the present sample, the WIAT score, an index of verbal ability, was significantly associated with the SS score in univariate and multivariate analyses. As a group, the individuals in our study clearly had problems engaging with school, with multiple suspensions and expulsions, and about a third left school without any qualification suggesting that as a group they might not perform well on cognitive testing. Surprisingly, however, the mean score of the reading test item from the WIAT II (Wechsler, 2001) for the sample was within the “average” range. This test is very similar in basic design to the National Adult Reading Test (Nelson and Willison, 1991) which is more widely used as a test resistant to the effects of brain damage to estimate “premorbid intelligence.” The precision of the reading test component from the WIAT II for estimation of “intelligence” is less well established. Thus, while we might have anticipated lower than average cognitive ability as a measure “explaining” lower than normative performance on OI, we do not have evidence to support that. On the other hand, the sample was selected based on the criteria of high BIS-11 score and previous studies have shown an association of low olfaction with impulsivity, a finding with which our findings are clearly consistent (Dileo et al., 2008; Herman et al., 2018; Brassard and Joyal, 2022). Thus, lower-than-expected olfactory performance in our sample, based on existing norms, might be explained partly by their high impulsivity, a behavioral characteristic influenced by OFC function/dysfunction. Whether the impulsivity itself, or underlying abnormalities of OFC suggested by it, explain the low olfactory scores is a matter we will consider more later.

The high prevalence of past TBI within the sample is also relevant to the low mean scores on the SS within the entire sample. Our analyses demonstrated a “dose effect,” namely an association of olfactory performance (and prevalence of hyposmia) with the severity of TBI based upon self-reported duration of LOC (Tables 3, 4, 5). Such findings are consistent with previous research linking TBI and olfactory dysfunction with a greater likelihood of olfactory impairment after more severe TBI (Ogawa and Rutka, 1999; Callahan and Hinkebein, 2002; Green et al., 2003; Sigurdardottir et al., 2010, 2016; Welge-Lussen et al., 2012; Gudziol et al., 2014; Schofield et al., 2014; Howell et al., 2018; Singh et al., 2018; Schneider et al., 2022). As indicated earlier, multiple mechanisms may account for the olfactory changes following TBI, including damage to frontal cortical structures such as OFC. OFC is a structure important both for olfaction identification and behavioral regulation; impulsivity, and most of the remaining correlates of olfactory performance that we found, can be considered as potentially reflective of those shared structural/functional affiliations.

Study criteria for the ReINVEST were designed to recruit men with a history of impulsive violence. The neurobiological basis for this syndrome is recognized to include an acquired heightened “threat response,” often during neurodevelopment, mediated by circuits within the brain which include the amygdala and the prefrontal cortex, including the OFC (Bertsch et al., 2020). Of the extrinsic factors known to confer risk for reactive (impulsive) aggression, adverse childhood events (ACE) (including sexual abuse) are especially toxic (Tomoda et al., 2009; Hanson et al., 2010; Van Harmelen et al., 2010; McCrory et al., 2011) and 31% of participants in our study reported such childhood sexual abuse. Reductions in OFC volumes have been consistently reported in the context of ACE and have also been shown in studies of violent individuals (Yang and Raine, 2010; Begemann et al., 2023). Other studies have shown positive correlations between OFC volume and olfactory identification scores (Seubert et al., 2012). Thus, multiple mechanisms—whether neurodevelopmental or due to damage or disease—that affect areas of OFC that engage both olfactory and behavioral regulatory networks may account for the associations we have identified.

An inverse association of olfaction and impulsivity has been found in a range of other settings, not necessarily violence-related. Thus, a previous study found olfactory identification deficits in war veterans with post-traumatic stress disorder (PTSD), which were also significant predictors of aggression (Dileo et al., 2008) and in a study of healthy volunteers, the two variables were associated (Herman et al., 2018). Given the known OFC affiliations of olfaction and impulsivity, and the susceptibility of this structure to damage in the context of TBI, common in offenders, it is unsurprising that we identified associations between those three variables (depicted in Table 5 and in regression analyses Tables 6, 7). Table 6 serves as an aid to conceptualizing the complex associations between impulsivity, TBI and offending, with olfactory performance serving as a proxy for OFC functioning. Inspection of that table indicates that more severe TBIs were associated with worse olfaction but without any difference in impulsivity, relative to milder TBI. One explanation for this dissociation of impulsivity with olfaction is that, in the context of TBI, olfactory impairments can be caused by sinus or peripheral olfactory pathway damage such as axonal shearing of olfactory fibers at cribriform plate that does not have direct behavioral/cognitive consequences. On the other hand, men who reported three or more TBIs were more impulsive (without having poorer olfaction), consistent with the notion that impulsivity is a risk factor for any and recurrent TBI. Neurobehavioral disturbances, such as high impulsivity, may exacerbate antisocial behavior, increasing the risk of criminality and recidivism and leading to TBI (Lane et al., 2017). Conversely, TBI can also lead to impulsivity and executive disorders, further emphasizing the complex interplay between these factors (Rochat et al., 2009; Kocka and Gagnon, 2014). Here, olfactory deficits might be present both before TBI—as correlates of OFC changes related to increased impulsivity—or after TBI—because of brain injury.

A further example of an association in our data that might reflect the measurement of function in topologically overlapping neural pathways is the positive association of SS score with social support scores suggesting that better olfactory function may be associated with stronger social support networks. The findings in relation to this variable were relatively weak—they were not apparent in the comparison of hyposmic and normosmic participants, and while significant in univariate analyses did not retain significance in the multivariate analyses (although *p* = 0.068 in the normal olfaction group). Previous studies indicate the importance of the frontal lobe, including OFC, for social functioning and an association of olfactory function (sensitivity) with measures of social connectedness has been demonstrated (Zou et al., 2016; Kwak et al., 2018). Social support has been identified as a protective factor against criminal behavior, highlighting the importance of understanding this relationship in offender populations (Cullen, 1994; Spohr et al., 2016). The finding that depressive symptoms were more frequent in the normosmic population, relative to hyosmics, was unexpected and contrary to previous research showing a link between olfactory dysfunction and depression (Naudin et al., 2012; Croy et al., 2014; Eliyan et al., 2020; Vance et al., 2023). That the symptoms were at most mild and we excluded individuals with active depression may partly explain this finding.

Our findings suggest that impaired olfaction, and therefore OI, may also serve as a marker for underlying neurobiological changes that contribute to behavioral disturbances, such as violence or those seen in PTSD or other anxiety-related disorders. The primary olfactory cortex and extended olfactory circuit, which are responsible for the neurobiology of olfaction, also share neural pathways and structures with the anxiety-fear system (Cortese et al., 2017). Finally, in line with our results, OI can serve as a proxy for TBI severity, given that TBI-related brain changes are also found in brain regions related to olfaction (Leutgeb et al., 2015).

The current study has several limitations. First, because participants were selected based upon a threshold level of impulsivity, the results of correlations between that measure and other characteristics are unlikely to reflect, in terms of the strength of association, what might be found within a more unbiased and representative population. Second, as this study is cross-sectional, no conclusions can be drawn with respect to causation. Third, all TBI data derives from history provided by the participants which is subject to a range of errors and biases, although we have previously reported evidence to suggest that offenders’ reports of past TBI have reasonable validity (Schofield et al., 2011).

We did not examine for peripheral olfactory disorders or other neuropathological disorders that could impact olfactory performance and our health survey did not include the details of the covariates of olfactory ability, such as medication and history of sinonasal injury; surgery was not controlled for. However, the health survey included the smoking history of all the participants and 83% of them were tobacco users (73% former and 10% current). The olfactory testing in this study relies on the verbal ability to identify the specific odor and a full measure of olfactory ability such as olfactory discrimination and olfactory acuity test would give a better understanding of the relationship between the current study’s sample and olfactory ability. We lacked a control group (i.e., non-impulsive non-offenders) which limits our ability to draw conclusions about the specificity of our findings with respect to the male and impulsive offender population and we lacked any brain imaging. Finally, because the study participants were characterized by elevated levels of impulsivity, we considered the possibility that impulsivity itself might affect olfactory performance, i.e., by participants not paying due attention to the testing process itself, and thereby making careless errors that were behavioral rather than olfactory perceptual. We hoped that the influence of impulsivity on the process of olfactory testing would be negligible, given the limited number of items in the short SS test.

In terms of practical applications, as we have discussed elsewhere, moving from the findings from group studies of olfaction and its correlates to considering the potential benefits of OI testing of individuals raises many questions and challenges (Ramaswamy and Schofield, 2022). The clearest benefit of OI is to detect olfactory impairments that would confer risk from failure to detect hazards such as smoke or spoiled food. If it were to be established by history that hyposmia/anosmia immediately followed a TBI, this would imply the probability of a more severe brain injury, especially if it was not accompanied by facial injuries (that might be responsible for a peripheral olfactory nerve injury). Interpreting the result of OI, at the individual level would mandate a minimum set of questions, many of which were not asked in the current study, including whether (and if so in what context) the sense of smell was lost or diminished. The history that anosmia or hyposmia closely followed a viral respiratory infection (a common scenario) would be vital information relative to the possibility that the olfactory changes are a marker of important OFC brain dysfunction (i.e., post-viral anosmia does not predict brain damage). Some people are unaware of olfactory impairment, however, and a negative history (e.g., an individual reports no history of TBI or of post-viral olfactory loss) is much more valuable than having no history at all. In the context of the criminal justice system, where individuals who enter the system have often previously sustained TBIs, a low performance on OI if done, might add weight to reports of a past severe TBI, or imply a severity when not reported or unknown. Finally, if anosmia were identified in an entrant to the criminal justice system, without any explanatory history, it might raise a flag to prompt further considerations as to the possibility of some relevant brain pathology. Although a very rare scenario, the olfactory loss can be due to a frontal tumor that may also cause behavioral changes (Snyder et al., 2000). In the context of current criminal justice system practices, where screening for important neuropsychiatric conditions tends to be limited (especially for cognition), weighing the cost benefits of OI against other competing needs would be challenging in the absence of more data and studies.

## 5. Conclusion

In conclusion, our study showed that, relative to population norms, impulsive violent offenders as a group performed more poorly on a test of OI. Their performance on this test was associated with other exposures (TBI, childhood sexual abuse) and other behavioral measures in patterns that would be consistent with olfaction serving as a proxy for OFC functioning. The possible role of olfactory testing within studies of offenders, or in their routine screening, warrants further consideration. In future studies we plan to examine whether olfactory test scores can aid in the prediction of offending, and whether they might contribute to the prediction of response to sertraline, in terms of offending reduction, within the ReINVEST study.

## Data availability statement

The raw data supporting the conclusions of this article will be made available by the authors, without undue reservation.

## Ethics statement

The studies involving humans were approved by these independent Ethics Committees: UNSW Human Research Ethics Committee (HREC) (HC11390 and HC17771), Aboriginal Health and Medical Research Council HREC (822/11), and Corrective Services NSW HREC (09/26576). Approval from the NSW Justice Health and Forensic Mental Health Network HREC (G8/14) will allow participants to continue the study while in detention. The studies were conducted in accordance with the local legislation and institutional requirements. The participants provided their written informed consent to participate in this study.

## Author contributions

VC: Conceptualization, Formal analysis, Writing – original draft. TB: Conceptualization, Funding acquisition, Methodology, Supervision, Writing – review & editing. BT: Data curation, Project administration, Writing – review & editing. KW: Funding acquisition, Writing – review & editing. PM: Funding acquisition, Writing – review & editing. LK: Project administration, Writing – review & editing. DG: Funding acquisition, Writing – review & editing. AE: Funding acquisition, Writing – review & editing. VG: Funding acquisition, Writing – review & editing. PS: Conceptualization, Funding acquisition, Methodology, Supervision, Writing – review & editing.

## Footnotes

1
https://www.nchecrsurveys.unsw.edu.au/shortfuses/
