# Supplementary material for: Neuropsychiatric correlates of olfactory identification and traumatic brain injury in a sample of impulsive violent offenders
Source: Front Psychol. 2023 Sep 29;14:1254574. doi: 10.3389/fpsyg.2023.1254574 (PMC10570745; doi:10.3389/fpsyg.2023.1254574)
Supplement: Supplementary file 1 [file Data_Sheet_1.docx]

**Supplementary Table 1: Participant psychological and functional measures by SS and without SS**

| **Variables** | **Overall (*n*=693)** | **No SS *(n*=208)** | **SS (*n*=485)** |  |
| --- | --- | --- | --- | --- |
|  | **mean (SD)** | **mean (SD)** | **mean (SD)** | ***P* value (a)** |
| **Barratt Impulsiveness Scale** | 85.00 (10.00) | 87.56 (9.50) | 84.51 (9.87) | **<0.001** |
| **Eysenck Impulsivity Questionnaire** |  |  |  |  |
| Impulsiveness | 13.60 (3.70) | 14.14 (3.22) | 13.34 (3.89) | **0.034** |
| Venturesomeness | 11.27 (3.07) | 11.57 (2.93) | 11.14 (3.13) | 0.103 |
| Empathy | 11.50 (3.60) | 11.54 (3.52) | 11.47 (3.68) | 0.982 |
|  |  |  |  |  |
| **Anger, Irritability, and Assault Questionnaire** |  |  |  |  |
| Irritability | 19.00 (6.00) | 18.89 (6.23) | 18.45 (5.99) | 0.289 |
| Anger Lability | 11.80 (4.90) | 11.54 (4.71) | 11.98 (4.98) | 0.338 |
| Direct Assault | 18.00 (7.00) | 18.28 (6.54) | 17.17 (7.00) | 0.057 |
| Verbal Assault | 17.00 (5.20) | 17.12 (5.57) | 16.96 (5.09) | 0.364 |
| Indirect Assault | 7.10 (3.60) | 6.80 (3.52) | 7.19 (3.59) | 0.254 |
| **Beck Depression Inventory** | 11.00 (9.00) | 10.87 (9.43) | 11.08 (8.26) | 0.304 |
| **Kessler Psychological Distress Scale** |  |  |  |  |
| k10 | 15.00 (9.00) | 14.25 (8.74) | 15.22 (8.79) | 0.150 |
| **Duke Social Support Scale** |  |  |  |  |
| Duke score | 24.20 (4.60) | 24.10 (4.56) | 24.20 (4.58) | 0.723 |
| **Quality of Life Short Form Questionnaire** |  |  |  |  |
| Physical component | 53.00 (7.00) | 52.27 (8.44) | 53.55 (6.87) | 0.189 |
| Mental component | 41.00 (12.00) | 41.81 (12.49) | 40.41 (12.33) | 0.144 |
| **State-Trait Anger Expression Inventory-2** |  |  |  |  |
| State Anger | 16.90 (4.50) | 17.29 (4.64) | 16.70 (4.50) | **0.014** |
| Trait Anger | 25.00 (7.00) | 24.92 (6.54) | 24.87 (6.62) | 0.688 |
| Anger Expression-out | 20.60 (5.00) | 20.58 (4.83) | 20.60 (5.04) | 0.748 |
| Anger expression-in | 19.20 (4.30) | 19.57 (4.35) | 19.09 (4.30) | 0.088 |
| Anger control-out | 17.40 (4.70) | 17.62 (5.00) | 17.37 (4.57) | 0.599 |
| Anger control-in | 18.40 (4.90) | 18.76 (5.36) | 18.18 (4.73) | 0.178 |
| Anger Expression-Index | 52.00 (14.00) | 51.77 (14.31) | 52.14 (13.53) | 0.859 |
| **Alcohol Use Disorders Identification Test** | 11.00 (9.00) | 9.23 (8.37) | 11.72 (8.71) | **<0.001** |
| **International Personality Disorder Examination** |  |  |  |  |
| Borderline | 323 (47%) | 104 (50%) | 219 (45%) | 0.241 (b) |
| Impulsive | 502 (72%) | 152 (73%) | 350 (72%) | 0.805 (b) |
| Dissocial | 320 (46%) | 113 (54%) | 207 (43%) | **0.005 (b)** |
| **Psychiatric assessment** |  |  |  |  |
| suicidal Ideation | 351 (51%) | 112 (54%) | 239 (49%) | 0.270 (b) |
| suicidal Attempt | 172 (25%) | 65 (31%) | 107 (22%) | **0.010 (b)** |
| self-harm or Injury | 169 (24%) | 56 (27%) | 113 (23%) | 0.309 (b) |
| sexual abuse | 277 (40%) | 125 (60%) | 125 (31%) | **<0.001 (b)** |
|  |  |  |  |  |
| **Substance Abuse (Current)** |  |  |  | 0.080 (b) |
| Yes | 570 (82%) | 163 (78%) | 407 (84%) |  |
| No | 123 (18%) | 45 (22%) | 78 (16%) |  |
|  |  |  |  |  |
| **Substance Abuse (Former)** |  |  |  | 0.433 (b) |
| Yes | 578 (83%) | 177 (85%) | 401 (83%) |  |
| No | 115 (17%) | 31 (15%) | 84 (17%) |  |

(a) One-way ANOVA (equal variance not assured; Bonferroni post Hoc test where significant);

(b) = Hosmer-Lemeshow Goodness of fit test; SD = Standard deviation. Bold values indicate statistical significance at 0.05 level.

**Supplementary Table 2: Demographic data by Sniffin Sticks category (n=693)**

| Variables | **Overall (*n*=693)** | **No SS (*n=*208)** | **SS (*n*=485)** |  |
| --- | --- | --- | --- | --- |
|  | ***n* (%)** | ***n* (%)** | ***n* (%)** | ***P* value (a)** |
|  |  |  |  |  |
| **Age in years** |  |  |  | 0.64 |
| 18-35 | 460 (66%) | 134 (64%) | 326 (67%) |  |
| 36-55 | 220 (32%) | 71 (34%) | 149 (31%) |  |
| >55 | 13 (1.9%) | 3 (1.4%) | 10 (2.1%) |  |
| **Ethnicity** |  |  |  | 0.972 |
| Not Abo/TSI | 589 (85%) | 179 (86%) | 410 (85%) |  |
| Aboriginal | 94 (14%) | 27 (13%) | 67 (14%) |  |
| Torres Strait Islander | 8 (1.2%) | 2 (1.0%) | 6 (1.2%) |  |
| Both Abo/TSI | 2 (0.3%) | 0 (0%) | 2 (0.4%) |  |
| **Marital Status** |  |  |  | 0.012 |
| Single (never married) | 365 (53%) | 117 (56%) | 248 (51%) |  |
| Regular partner | 170 (25%) | 36 (17%) | 134 (28%) |  |
| Married | 113 (16%) | 45 (22%) | 68 (14%) |  |
| Separated | 33 (4.8%) | 8 (3.8%) | 25 (5.2%) |  |
| Divorced | 11 (1.6%) | 2 (1.0%) | 9 (1.9%) |  |
| NA | 1 (0.1%) | 0 (0%) | 1 (0.2%) |  |
| **Number of Children, mean (SD)** | 1.76 (2.00) | 1.86 (2.09) | 1.72 (1.97) | 0.403 (c) |
| **Age at leaving school, mean (SD)** | 15.66 (1.61) | 15.60 (1.31) | 15.69 (1.72) | 0.939 (c) |
| **Number of schools changed before dropping out, mean (SD)** | 4.51 (4.85) | 4.69 (3.09) | 4.43 (5.43) | 0.303 (c) |
| **Number of times suspended from school, mean (SD)** | 11 (18) | 12.62 (15.77) | 10.88 (19.20) | **0.007 (c)** |
| **Number of times expelled from school, mean (SD)** | 2.15 (2.80) | 2.25 (3.33) | 2.11 (2.53) | 0.516 (c) |
|  |  |  |  |  |
| **WIAT score, mean (SD)** | 116 (16) | 116.36 (14.72) | 116.35 (16.30) | 0.216 (c) |
|  |  |  |  |  |
| **Education** |  |  |  | 0.702 |
| Never attended school | 4 (0.6%) | 1 (0.5%) | 3 (0.6%) |  |
| Completed primary school only | 14 (2.0%) | 2 (1.0%) | 12 (2.5%) |  |
| Left school with no qualification | 226 (33%) | 70 (34%) | 156 (32%) |  |
| School certificate | 251 (36%) | 74 (36%) | 177 (36%) |  |
| HSC/VCE/Leaving certificate | 72 (10%) | 20 (9.6%) | 52 (11%) |  |
| College certificate/diploma | 24 (3.5%) | 10 (4.8%) | 14 (2.9%) |  |
| Technical or Trade qualification | 86 (12%) | 25 (12%) | 61 (13%) |  |
| Degree/tertiary education | 13 (1.9%) | 4 (1.9%) | 9 (1.9%) |  |
| NA | 3 (0.4%) | 2 (1.0%) | 1 (0.2%) |  |
| **Employment** |  |  |  | **0.023** |
| Unemployed for at least 6 months | 286 (41%) | 100 (48%) | 186 (38%) |  |
| Employed within last 6 months | 405 (58%) | 107 (51%) | 298 (61%) |  |
| NA | 2 (0.3%) | 1 (0.5%) | 1 (0.2%) |  |
| **Accommodation** |  |  |  | 0.666 |
| Live alone | 92 (13%) | 29 (14%) | 63 (13%) |  |
| Partner | 188 (27%) | 57 (27%) | 131 (27%) |  |
| Mother/father | 193 (28%) | 54 (26%) | 139 (29%) |  |
| Mother/father and partner | 14 (2.0%) | 2 (1.0%) | 12 (2.5%) |  |
| Other relatives | 202 (29%) | 64 (31%) | 138 (28%) |  |
| NA | 4 (0.6%) | 2 (1.0%) | 2 (0.4%) |  |
| **Have you ever attended any special schools/classes?** |  |  |  | 0.395 |
| No | 452 (66%) | 140 (68%) | 312 (65%) |  |
| Yes | 237 (34%) | 66 (32%) | 171 (35%) |  |
| **Have you ever been expelled from a school?** |  |  |  | 0.398 (b) |
| No | 410 (59%) | 118 (57%) | 292 (60%) |  |
| Yes | 280 (41%) | 89 (43%) | 191 (40%) |  |
| **Have you ever been suspended from a school?** |  |  |  | 0.164 |
| No | 167 (24%) | 43 (21%) | 124 (26%) |  |
| Yes | 522 (76%) | 164 (79%) | 358 (74%) |  |

(a) = Hosmer-Lemeshow Goodness of fit test; (b) = Fisher's exact test; (c) One-way ANOVA (equal variance not assured; Bonferroni post Hoc test where significant); SD = Standard deviation. Bold values indicate statistical significance at 0.05 level.
